# The Immuno-Dynamics of Conflict Intervention in Social Systems

**DOI:** 10.1371/journal.pone.0022709

**Published:** 2011-08-24

**Authors:** David C. Krakauer, Karen Page, Jessica Flack

**Affiliations:** 1 Santa Fe Institute, Santa Fe, New Mexico, United States of America; 2 Department of Mathematics, University College London, London, United Kingdom; 3 Yerkes National Primate Research Center, Atlanta, Georgia, United States of America; University of Maribor, Slovenia

## Abstract

We present statistical evidence and dynamical models for the management of conflict and a division of labor (task specialization) in a primate society. Two broad intervention strategy classes are observed– a dyadic strategy – pacifying interventions, and a triadic strategy –policing interventions. These strategies, their respective degrees of specialization, and their consequences for conflict dynamics can be captured through empirically-grounded mathematical models inspired by immuno-dynamics. The spread of aggression, analogous to the proliferation of pathogens, is an epidemiological problem. We show analytically and computationally that policing is an *efficient* strategy as it requires only a small proportion of a population to police to reduce conflict contagion. Policing, but not pacifying, is capable of effectively eliminating conflict. These results suggest that despite implementation differences there might be universal features of conflict management mechanisms for reducing contagion-like dynamics that apply across biological and social levels. Our analyses further suggest that it can be profitable to conceive of conflict management strategies at the behavioral level as mechanisms of social immunity.

## Introduction

In large societies of individuals or cells, sophisticated regulatory mechanisms are required to control conflict and promote coordination [1]–[7]. These conflict management mechanisms can involve specialization through to a full division of labor [8], [9], or the creation of social norms that reinforce roles and behavioral patterns [10]. The organism has a dedicated suite of conflict management mechanisms, including an immune system, that regulates cell-cell and cell-pathogen interactions [11]–[13]. Beyond research on policing in social insects [14], [15] and punishment in human societies (e.g. [15]–[17]), relatively little is known about conflict management dynamics in animal societies. The common presumption that organismal conflicts of interest promote a tragedy of the commons has generated low expectations for group-level regulation.

Nonetheless, conflicts in animal societies lead to fights, and these do not typically overwhelm the group or result in severe injury or death as there are a variety of mechanisms animals use for resolving disputes or mitigating the effects of aggression [2]. In some societies conflicts are managed by other individuals in the group[2], [6]. One management strategy third-parties use is to calm agitated individuals (see box 13.1 in [18]), forestalling aggression. These interventions are called *pacifying* interventions. A second strategy is an impartial intervention –a *policing* intervention– in which all conflict participants are targeted indiscriminately by a third-party through aggression, or through the implicit threat of aggression inherent in an approach by the third-party [6]. Both pacifying and policing interventions can cause the fight to terminate and/or aggression to dissipate [6].

In this paper we use an immuno-dynamics modeling approach [19], [20] to explore the consequences of third-party conflict management for the contagion of aggression. Our primary goal is to determine whether policing and pacifying interventions have different effects on aggression dynamics and hence different mechanistic benefits. A second, more ambitious goal is to ask why, as with cellular immunity, we observe in social systems triadic as well as dyadic strategies for managing conflict. We use the macaque genus, a model system for social evolution [21], [22], to develop the theory, working with a data set describing conflict dynamics collected from a large pigtailed macaque (Macaca nemestrina) society housed at the Yerkes National Primate Research Center (Methods 1).

### A multi-scale immuno-dynamics approach

**Figure 1:**
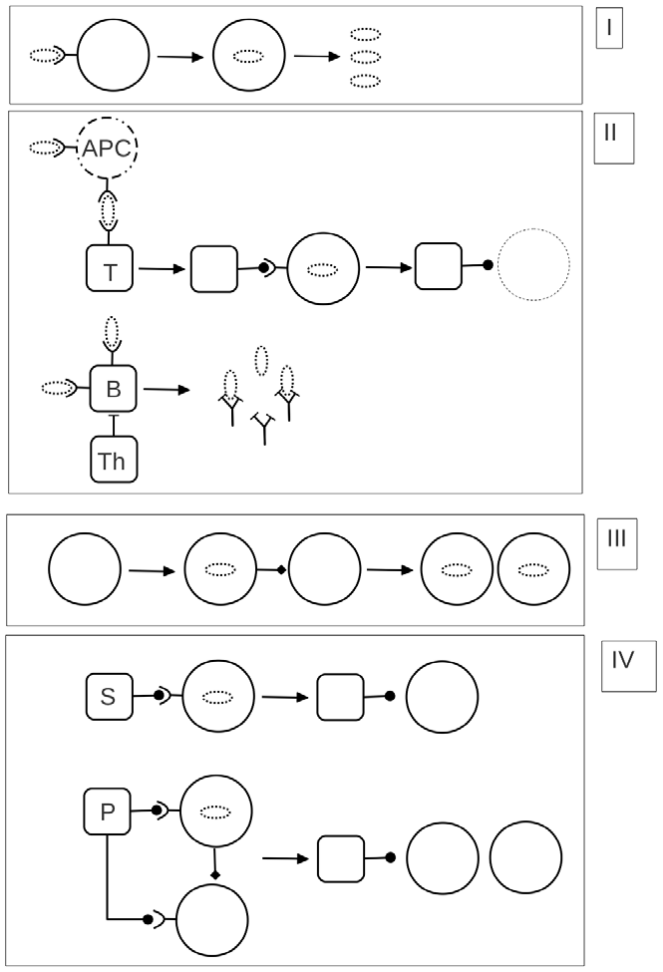
Comparison of the structure of cellular and social immunity. The top two panels illustrate cellular infection and immunity. The bottom two panels illustrate social infectivity and social immunity. (I) Pathogens infect cells and thereby proliferate. (II) Naive T cells are presented with antigen epitopes by antigen presenting cells (APC), inducing them to target infected cells and eliminate them. B cells sequester antigen and are induced to generate antibodies to these by T helper cells. (III) Individuals become aggressive and by direct contact with others redirect aggression infectiously. (IV) Pacifiers (S) engage with aggressive individuals, calming them down thereby preventing redirection. Police (P) intervene directly into conflicts, resolving disputes and returning combatants to the passive state. In animal conflict it is a state of behavior that is transmitted that arises spontaneously from within the population.

In the case of policing in animal societies, the aggressive dyad or fight-complex, is targeted, eliminated, and resolved into peaceful or “passive” individuals. As illustrated in Fig. 1., the triadic character of policing interventions (P) is roughly comparable to the action of T cells that identify infected cells (a complex of pathogen and host cell) and eliminate them from a population. During a pacifying intervention an aggressive or agitated individual is targeted and resolved into a peaceful state: Pacifiers (S) identify aggressive individuals on route to fight, or while the fight momentarily abates, and through direct interaction, induce them to transform to a passive state (Fig. 1 IV upper schematic). Pacifying interventions are comparable to the behavior of antibodies engaging pairwise with pathogens to prevent infection of susceptible cells.

Without an immune response infection threatens to exponentially encompass a population of susceptible cells (Fig. 1 I). The T cell response requires activation of naive T cells by antigen presenting cells. The T cells are then able to recognize “foreign” antigens on the infected cell surface and remove these cells clearing the infection (Fig. 1 II upper schematic). Homeostatic regulation of the cell population restores depleted cells. B cells bind antigen directly, and through interaction with helper T cells, generate antibody to antigen, leading to antigen clearance prior to cellular infection (Fig. 1 II lower schematic). Similarly in the absence of social immunity aggression in an animal society threatens to exponentially encompass a group through behavioral redirection of aggression (Fig. 1 III) [23], [24].

Unlike cellular immunity, however, neither policing nor pacifying interventions kills the target. Instead these strategies terminate deleterious behavioral patterns, transforming the target and keeping the population size constant. Neither behavioral strategy requires “fourth” party activation of the conflict management mechanism –hence there is no apparent social immune analog to cellular immune priming. In contrast to cellular immunity, the effectiveness of policing and pacifying interventions is not assured, as effectiveness depends in part on characteristics of the individual performing the intervention, on the character of the conflict itself, and on properties of the conflict time-series [6], [24].

Adaptive immune systems possess the property of antigen mediated clonal selection and expansion. In animal societies, in contrast, passive individuals can spontaneously adopt a policing strategy and remain in that state until the number of aggressive individuals decreases to some threshold value. Another important difference is that within the biological immune system, pathogens form a population independent from immune cells, whereas behavioral conflict management strategies are implemented by individuals belonging to the same group as the conflict participants. Hence functional constraints on conflict management due to partially aligned interests among group members are likely more significant in social systems than in the case of pathogen control.

However, in mechanical terms the fundamental pathogenic property is the ability to transform the state of a cell, increase cellular rates of mortality and proliferate through a population. These are also properties of aggression that can be thought of as a transmissible state of behavior. Proliferation of aggression leads to larger fights and an increased probability of individual mortality [23], [24]. The contagion property of both pathogens and aggression, coupled to similar management mechanics instantiated in dyadic and triadic interactions, suggests that comparison are warranted despite critical differences in implementation.

In the empirical section of the Results, we further describe the mechanics of policing and pacifying interventions in our pigtailed macaque study system. In the theory section of the Results we develop empirically-grounded immuno-dynamics models to explore the implications of these alternative strategies for containing aggression.

## Results

### Empirical description of pacifying and policing in pigtailed macaques

In previous work [6], it was shown empirically that pigtailed macaques use both policing and pacifying interventions. It was also shown that the frequency distribution of policing interventions is heavy-tailed. The frequency distribution of pacifying interventions on the other hand is normal. This difference suggests that there might be a proto-division of labor for policing interventions but not for pacifying interventions. Here we determine whether this is the case.

We define a proto-division of labor as specialization on a group beneficial task by a subset of components in the absence of complementary specialization by a second subset. A full division of labor minimally involves two subsets, each specializing on complementary tasks [25]. Role specialization, defined here as either individual, age-sex class, or other subgroup specific strategy sets, is a foundational assumption of game dynamics yet is rarely empirically evaluated outside the study of cellular immunity or social insect societies. Role specialization on strategy sets can be operationalized statistically.

An individual is said to specialize on *conflict management generally* if its policing (POL) frequency is 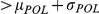 AND pacifying (PAC) frequency is 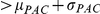, where 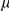 is the sample mean and 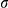 denotes one standard deviation about the mean. *Policing specialization* is POL 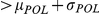 and PAC 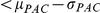. *Pacifying specialization* is POL 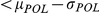 and PAC 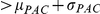 (unless otherwise noted, all empirical analyses in this paper use corrected frequency data, in which an individual's frequency is equal to a measured deviation from an expected score, see Methods 1). Note that whereas the distribution of pacifying interventions is normal, the distribution of policing interventions is roughly heavy-tailed. Given that we are interested in evidence for concerted deviations from the average behavior of *all* individuals in the group, including those in the tail, we operationalize specialization with respect to the standard deviation of the policing and pacifying distributions regardless of the form of those distributions (e.g. normal, heavy tailed, etc).

**Figure 2:**
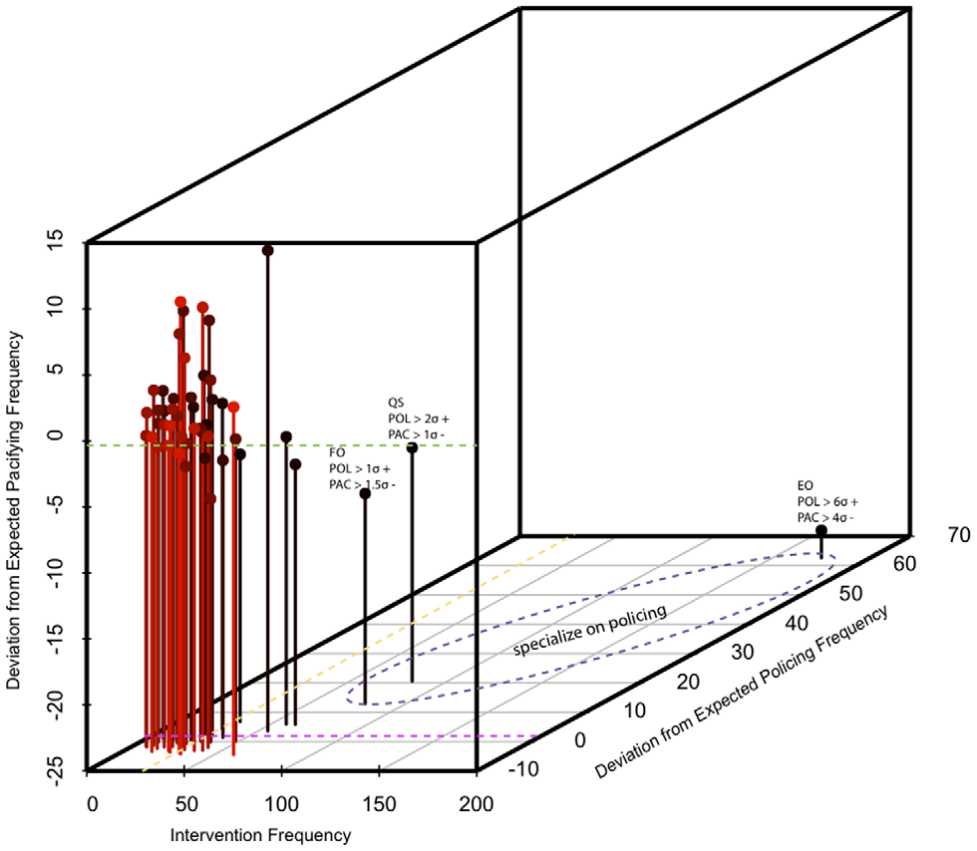
Specialization on policing produces a proto-division of labor in a macaque society. Only three individuals, EO, QS, and FO, enclosed by the dashed purple ellipse, perform policing at a minimum of one standard deviation greater than the mean deviation from expected frequencies for the population (DEF; see text for definition) and pacifying at one standard deviation less than the mean DEF. EO's policing frequency is six standard deviations greater than the mean DEF. The dashed green line indicates the mean deviation from expected pacifying frequency (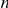 = 48). The dashed fuscia line indicates the mean deviation from expected policing frequency (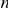 = 48). The dashed yellow line indicates the mean frequency of intervention performed by the 48 socially-mature individuals. The bar color is graded from red to black to make individual differences easier to see. The patchy distribution of the state space supports the interpretation of behavior in terms of statistically-defined strategy classes.

Considering the 48 socially mature animals in the group, we find no evidence for general conflict management specialization and no evidence for pacifying specialization. Only three adult males, EO, QS, FO, exhibit policing specialization Fig. 2. These three individuals account for 39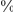 of the 477 policing interventions but only 10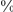 of the 304 pacifying interventions. The remaining 45 socially-mature group members show no preference for either policing or pacifying, performing as many policing as pacifying interventions (deviation from expected frequencies (DEF), policing data are non-normal, 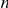 = 45, Wilcoxon signed rank test, 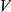 = 460, 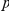 = .52). Elsewhere we have shown that the distribution of social power (degree of consensus among group members that an individual can use force successfully during fights), by modulating the cost of social interaction, influences individual strategy choice [6], [26], [27]. In our study group the power distribution is not significantly different than lognormal (log-transformed (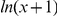) data, Lilliefors KS test, 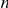 = 48, 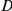 = 0.123, 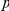 = .08). The three individuals specializing on policing (henceforth, “the policers”) occupy the tail of this power distribution and respectively have 24.29, 8.85 and 7.82 times more power than the average individual among the remaining 45.

Approaching conflicts, required for both pacifying and policing interventions, is costly because conflict participants frequently redirect aggression to interveners [6], [28]. The cost of pacifying and policing interventions can be measured as aggression or threat received in response to intervention (Methods 1). We find that cost decreases with increasing power for policing but not pacifying interventions (data include policers and the 42 of 45 non-policers who perform both pacifying and policing interventions, DEF POL: data nonnormal, 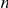 = 45, Kendall's Rank Correlation: 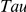 = −0.26, 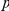 = 0.01, DEF PAC: data nonnormal, 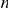 = 45, 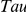 = −0.08, 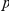 = .48).

We find no significant difference between cost of pacifying and cost of policing for the 42 non-policers (deviation from expected cost (DEC), data are non-normal, 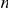 = 42, Wilcoxon signed rank test, 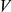 = 375, 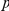 = 0.35). The cost paid by policers for policing is two orders of magnitude less than the average deviation from expected cost paid by the other 42 animals (DEC: 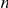 = 45, 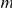 = −1.36, 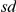 = 5.11, EO = −30.30, QS = −6.59, FO = −6.691, remaining 42 individuals: 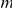 = −0.32, 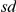 = 2.25). EO was not observed to receive aggression in response to any of his 104 policing interventions.

The policers pay a slightly higher cost than expected for pacifying (DEC: 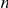 = 45, 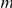 = 0.097, 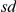 = 1.63, EO = −1.34, QS = 0.04, FO = 2.32, remaining 42 individuals, 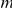 = 0.06, 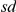 = 1.66), but this cost is still negligible as they receive threats in response to fewer than 5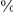 of their pacifying interventions and never receive contact aggression.

We find that the effectiveness –the ability to terminate a fight or reduce the severity of aggression (Methods 1) – of policing, but not pacifying, increases with increasing power (data include the three policers and the 42 nonpolicers who perform both intervention types, DEF POL: data nonnormal, 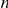 = 45, Kendall's Rank Correlation: 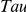 = 0.34, 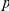 = 0.001, DEF PAC: data nonnormal, 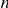 = 45, 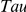 = 0.19, 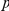 = .07). The policers are more than four orders of magnitude more effective at policing than the remaining 42 individuals (DEF: all 45 individuals, 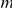 = 1.9, 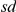 = 9.61, EO = 61.64, QS = 15.07, FO = 8.90, remaining 42 individuals, 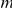 = .0005, 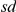 = 1.67).

The policers are 11 times more effective than expected at breaking up or reducing the intensity of fights using policing interventions than they are when using pacifying interventions (DEF POL: EO = 61.64, QS = 15.07, FO = 8.90, DEF PAC: EO = 4.30, QS = 1.50, FO = 2.15). The remaining 42 socially-mature group members perform as many effective policing as effective pacifying interventions (DEF, data are non-normal, 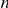 = 42, Wilcoxon signed rank test, 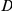 = 474, 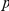 = .78).

To summarize, the data indicate that a small subset of the group performs policing, everyone engages in pacifying, and policing is better than pacifying at controlling the escalation of aggression when the policers are powerful. The effectiveness and cost of policing appear to depend on relative power in a heavy-tailed power distribution. Hence a power-based *state dependence* supports specialization on policing but does not influence pacifying. We find no evidence suggesting that pacifying is state dependent.

### Social immuno-dynamics modeling results

We have explored elsewhere why a high variance distribution of power is required to support policing [6], [26]. We and others have also considered how conflict management mechanisms such as policing evolve [3], [5], [15], [26]. We seek an ontogenetic explanation for how policing, when performed by few individuals, can effectively control conflict or reduce its frequency in social groups. And, why pacifying strategies, performed by many individuals, are less effective. If we are able to reproduce these conflict management patterns, we shall have succeeded in accounting for the division of labor in policing, and the widespread, undifferentiated, adoption of pacifying. This will provide the beginnings of an account for diverse forms of conflict management at the social level, and the grounds for a more informed comparison with the control of contagion among populations of cells.

We develop two classes of models –one for pacifying and one for policing. We explore how the degree of specialization on a management strategy influences conflict dynamics (for a review of this approach [19], [20]). Degree of specialization in these models is operationalized as the proportion of individuals in the group performing conflict management.

In the pacification model, we assume a population of passive individuals 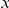 that spontaneously become aggressive 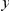 at a rate 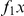 (Methods 2). Once in an aggressive state, these are capable of “infecting” further individuals through social contagion inducing them to become aggressive. From the resulting aggressive dyad or complex 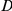, emerge two aggressive individuals. Monitoring is performed by a population of individuals – conflict managers 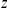 – who identify aggressive states prior to the formation of the complex and form an intervention dyad 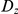. This resolves into a single manager and pacified individual. In this model, conflict managers have no influence over conflicts that have already begun. The initial conditions corresponding to the start of observations of behavior are, 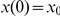, 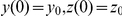 and 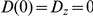.

**Figure 3:**
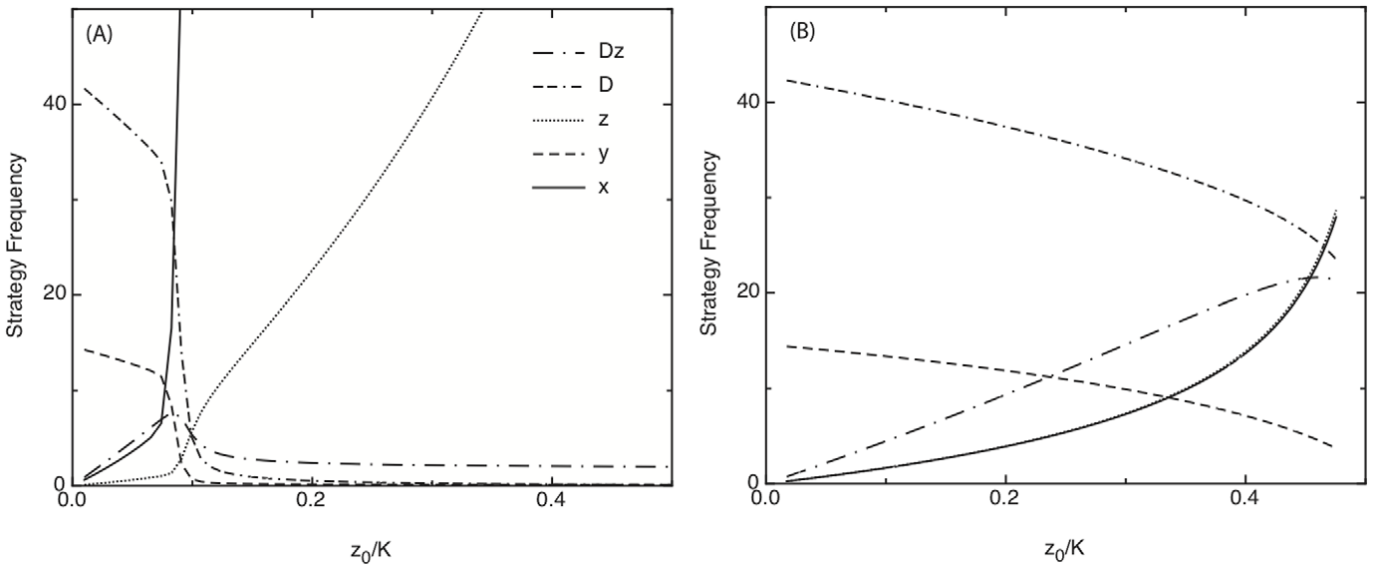
Steady state frequencies of aggressive individuals 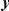, passive individuals 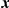, fighting pairs, 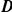, and police 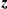 as a function of the proportion of policing individuals 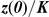. Assuming policing interventions (A) we observe a threshold value of police above which conflict is effectively eradicated. Under pacifying interventions (B), conflict declines monotonically in the proportion of pacifiers. For these figures we have used parameter values: 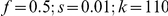. The parametric sensitivity of these results are indicated analytically in the text.

In Figure 3B, we illustrate the steady state frequencies of the each of the state variables as a function of the proportion of individuals in the population assuming a pacifying, conflict management role. We find that the total number of fights declines monotonically with increased pacifying as do the number of aggressive individuals. For a large decrease in aggression, there needs to be a concomitant large increase in the proportion of individuals in the population assuming the pacifier role.

Given that pacifiers exist in only two states, and their total number is assumed to be conserved, we make the observation that,
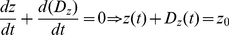
(1)This allows us to express the state variable 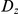 in terms of 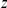 and the initial number of pacifiers at the start of observations, 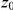. Furthermore, we assume that the total population size remains constant,
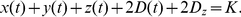
(2)This allows us to express the fight complex 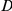 in terms of 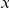, 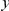 and 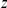 and two constants:
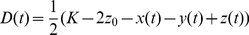
(3)We minimize the number of parameters by assuming that there are three time scales in the dynamics. A time scale at which fights are initiated and monitored, a second time scale at which fights are resolved, and a very slow time scale at which aggression emerges. Thus 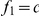 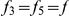 and 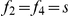. These assumptions allow us to write down a 3-dimensional dynamical system (rather than 5-dimensional), that describes the conflict management dynamics.
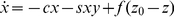
(4)

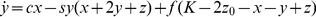
(5)

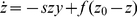
(6)


As per our assumptions: 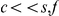, and furthermore, that the population size is significantly greater than the number of police, 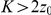. We find that there are two steady states. The first steady state is at 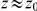, 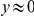, 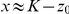, 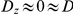. This state is always unstable. The other steady state is at 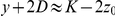, 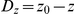 and 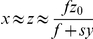, where 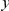 is approximately the unique positive solution to 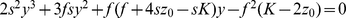. Thus if there are few police, 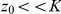, the population is made up largely of aggressors (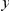) and “fighters” (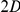). For fixed values of the remaining parameters, the steady state values of 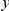 and 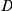 are monotonically decreasing in 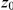.

In the policing model, the conflict managers, called policers 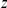, do not target aggressives 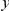 before they engage in conflict as in the antibody model, but resolve disputes directly, by intervening and eliminating fights between pairs forming in this case a triadic variable 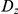 composed of both the complex 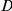 and the policer 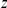. As before, the conflict managers exist in two states, and their total number is conserved:
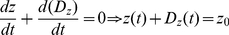
(7)The total population size is constant,
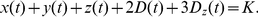
(8)Thus
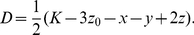
(9)


We assume three timescales, with 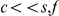 and 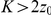. In addition, we assume 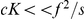.
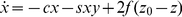
(10)

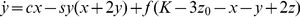
(11)

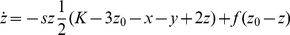
(12)


This dynamic has a steady state with 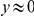. In this case, however, for critical values of the parameter set, this state is stable. In particular the state has 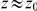, 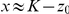 and 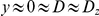 and is stable iff 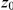 is greater than approximately 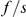. Thus we have a state in which there are almost no aggressors or fights being stable provided there are initially more than a threshold number of policers. This threshold declines as the duration of fights increases and as the interaction rate (which is both the rate at which fights are initiated and the rate at which police intervene in fights) increases. When these values are high there are few free aggressors. Interestingly the policing threshold appears to be independent of the population size 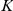, provided that this size is not too large (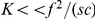). Thus unlike in the B cell model, the T cell model is capable of leading to stable societies in which there is no unrest with only small rates of conflict management. This modeling finding is consistent with the data from our study group in which 17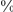 of the 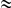
1100 conflicts observed received effective policing interventions. This level of policing has been shown empirically in a behavioral knockout experiment to be sufficient to reduce general levels of aggression [23].

**Figure 4:**
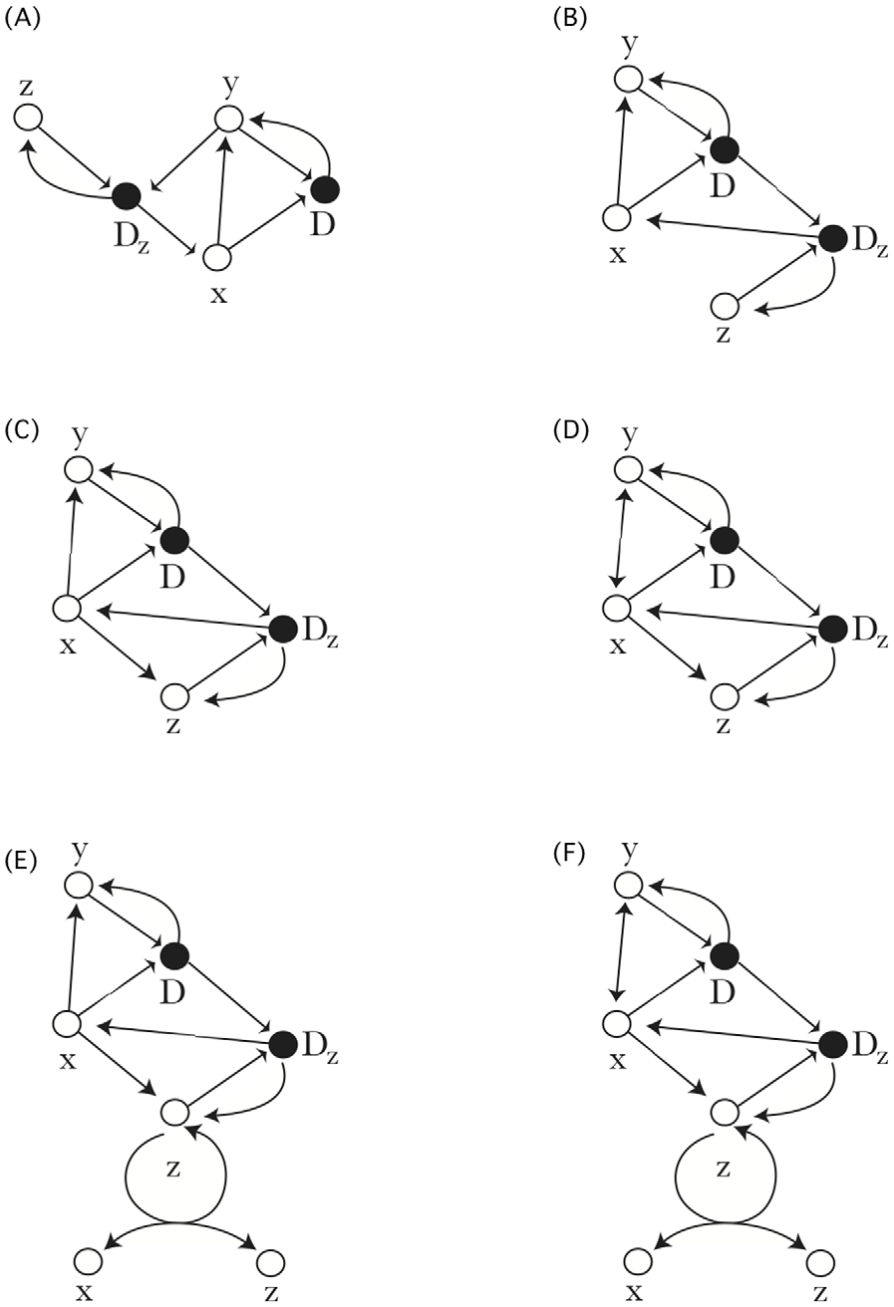
Conflict networks illustrated as reaction graphs. Open nodes are single individuals. Closed nodes are groups of 2 or more individuals. Directed edges represent transformations but not stoichiometry. Merging input edges into a single closed node corresponds to the formation of a complex (dyad or triad). Cycles represent transformations that yield one starting state and one alternative. The six conflict networks are: (A) B-cell inspired pacification. (B) T-cell inspired policing. (C) Spontaneous policing. (D) Temporary aggression. (E) Conditional Policing. (F) Conditional, Temporary Policing. Hence in (A) 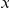 becomes aggressive and transforms to 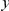. The aggressive individual 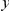 interacts with 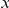 to form a fight 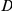 which then resolves into aggressive individuals 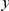. The strategy 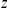 interacts with 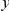 to form the pacifying dyad 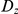 which then resolves into 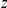 and 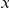. In the most complicated example (F), 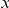 becomes aggressive and transforms to 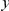, and 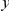 can revert to 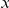. The aggressive individual 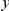 interacts with 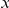 to form a fight 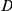 which then resolves into aggressive individuals 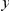. The strategy 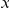 can spontaneously transform into 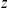. The strategy 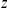 can interact with 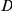 to form the triadic complex 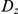, which resolves into 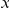 and 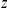. he strategy 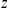 can spontaneously transform into 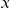.

We consider a larger family of models expanding on our basic policing and pacifying structures to cover and analyze a richer space of strategic permutations. These are illustrated using conflict reaction graphs in Figure 4 (full mathematical description in Methods). These include cases in which passives can transform into police (spontaneous policing); aggressives can transform into the passive state spontaneously (temporary aggression), and where the policers switch to non-policing when policing is common (conditional policing – negative frequency dependence). The results of all of these models are summarized in Table 1. Each model possesses multiple stationary states (a maximum of three) and we indicate those that are stable. Of greatest social interest are those strategies where multiple equilibria exist. These are models that allow transitions between strategy classes, such as switching from a policer to a pacifier. In the case where pacifiers become police spontaneously at a low rate, and where policing interventions can fail inducing the police to become aggressive, this results in a solution with multiple equilibria. These include a population that engages in chronic violence (only aggressive individuals and aggressive dyads exist), a ‘civil-solution’ in which policing effectively controls aggression and passive individuals dominate, and a ‘police-state’ in which the entire population is driven to become police. The most harmonious case arises when we assume that aggressive individuals can transform spontaneously to passive individuals. In this case, the combination of policing and a tendency towards peace, generates a population with minimal aggression, dominated by a few police and many passives.

**Table 1:** The composition of multiple, stable equilibria under six models of third party intervention.

Model	Equilibrium 1	Equilibrium 2	Equilibrium 3
1. Pacifying	x,y,z,D,D_z_	–	–
2. Policing	x,y,z,D,D_z_	z,x	–
3. Spontaneous policing	y,D	x,z	z
4. Temporary aggression	x	Z	–
5. Conditional police	x	y,D	–
6. Conditional, temporary	x,y	–	–

Models represent variation in pacifying and policing strategies. Models are described in the text and full mathematical details are provided in Methods 2. Those configurations for which there exist stable solutions are marked by the variables present in their locally stable equilibria. Empty columns (marked by a dash) are not stable or do not exist. Hence in the pacifying model (model 1) all strategies are stable and present at equilibrium. In the model of spontaneous policing (model 3) there are three stable equilibria, a violent equilibrium in which there are only fights and aggressive individuals present, a civil equilibrium in where there are passive individuals and police, and a police-state equilibrium in which all individuals become police.

We also consider models that allow for a form of proto-clonal expansion through negative frequency dependence. In the class of strategies that we refer to as *conditional*, police transform into a passive state when the frequency of police in the population is high. Hence when two police meet, one will transform into a passive. This generates two stable equilibria. One, the ‘Utopian equilibrium’, in which the entire population becomes passive. And another “Dystopian equilibrium” in which the population descends into violence.

These more complex models of conflict management illustrate the double-edged sword of policing. If conditions are favorable, then policing can be very effective at reducing or eliminating conflict, with a small population of police. But if policing can fail or policing ceases in response to the presence of others (a form of free-loading), policing can lead to deleterious outcomes in which populations become police states -everyone polices - or violence becomes chronic and ubiquitous.

## Discussion

We have investigated mechanisms that minimize the contagion of deleterious conflict in a social system. We described the mechanics of two fundamental classes of conflict management mechanism empirically, using data collected from a primate society model system, and mathematically, using dynamical models inspired by the structure of immune systems. We observe a dyadic (two-way) class in which individuals preempt aggression thereby preventing the contagion of aggression (pacifying), and a triadic class (three way) in which individuals directly manage ongoing conflicts (policing), minimizing the redirection and propogation of aggression.

We observe empirically that there is no individual specialization for pacification behavior. Pacification is performed by many individuals. In our model simulations and analysis aggression declines monotonically with an increasing frequency of pacification. We observe empirically that there is however specialization by a few individuals for policing interventions. In our simulation and analytical results policing but not pacifying is capable of almost completely eliminating conflict, and does so above a critical-threshold proportion of police. These results are consistent with a previous experimental study in which it was found that pacifying interventions alone were not sufficient to maintain low levels of aggression [23].

When policing interactions can fail – aggravating aggression and inducing non-policers to switch to policing in compensation – multiple stable equilibria are observed (Model 3 Table 1). The population can either occupy a highly aggressive state, the population of policers can grow to take over the population in order to control aggression, or policing and passive individuals can coexist at comparable numbers. The solutions are determined by the balance between spontaneous policing and the corruption of police following failed interventions. Our empirical data suggest that policing is a *relative* state dependent strategy, in so far as it appears to require a high variance in the distribution of power, or some other analog measure of resource disparity, to arise. Hence the second and third of the equilibrium states of this model are unlikely to be realized as these states assume that many individuals assume a policing role - albeit somewhat ineffectively. The first equilibrium state –a transition to high aggression when policing fails– is consistent with experimental findings showing that when policing is disabled, aggression increases, and hence that effective policing is critical to preventing social destabilization in systems in which policing is the primary conflict management mechanism [7], [23].

Finally, we have shown analytically that policing (T cell strategy) is the more *efficient* strategy, as conflict can be eliminated with only a small proportion of policers. We find no such threshold for pacifying interventions, nor is there any empirical support suggesting such a threshold. The advantage of pacifying interventions is that they are not dependent on power but can be used by anyone, and thus might provide a first line of defense against contagion of aggression in the absence of complex social structure. More generally, our results here and in previous work [6] suggest that social structure is a constraint on conflict management, with different distributions of power, for example, favoring different conflict management mechanisms.

### Comparison to Cellular Immunity

The immuno-dynamics approach and its results reveal critical similarities between social and cellular immunity. Both can be thought of as evolved mechanisms for minimizing the costs of contagion. Similarities include: (1) Dedicated agents adapted to preventing propagation of deleterious or dangerous states. (2) An ability to recognize, engage and clear deleterious factors. (3) A pairwise mechanism and a triadic mechanism. (4) Role differentiation for some aspects of conflict control. (5) Thresholds in the response associated with clearance of danger.

Critical differences include: (1) Non-destructive interactions in the social mechanisms and destructive mechanisms in the cellular mechanisms. (2) Direct activation in the social case and fourth-party mediated activation in the cellular case. (3) Selective proliferation of distinct cell-types in the cellular case, and a steady state response or generic proliferation in the social case. (4) An ability to assume both pacifying and policing roles in the social case, but with only a subpopulation effective in the policing role. (5) Differentiation into management roles in primate social systems acquired through a comparatively fast learning mechanism rather than genetic mechanisms such as somatic hypermutation.

In much the same way that immuno-dynamics grew out of the application of epidemic models to the cells of a single individual, and prion dynamics grew from immuno-dynamics in the absence pathogens assuming only protein mis-folding, here we have considered the immuno-dynamics of a state of behavior without an extrinsic pathogen-like entity. As with prions in which misfolded proteins induce further misfolding, aggressive individuals induce further aggression. The key analogy across all of these systems is our ability to describe them using dynamical systems that take account of contagion and evolved mechanisms of mitigation.

### Evolutionary-ecological Issues

In emphasizing mechanisms for the containment of aggression within a single generation, the approach we have adopted is more akin to ecological models of microbial infection and clearance than to evolutionary models in social evolution of punishment (e.g. [15]–[17]) and policing (e.g. [3], [5], [15]). The goals of models of punishment and policing are to identify optimal or stable parameter values that through fixed payoffs facilitate cooperative evolution. Punishment is typically defined functionally, with the term punishment applied when an individual, at a cost to itself, inflicts a cost on another individual for failing to cooperate. Policing is typically defined as the repression of competition, and sometimes mechanistically as impartial or indiscriminate conflict intervention that can lead to the repression of competition, as in this paper. Much work on punishment assumes policing has the same basic payoff structure to punishment and hence is a subclass of punishment.

One advantage of studying ecological effects is that doing so can reveal a complex strategy space lurking behind functional assumptions. It is well known in the empirical community that there are multiple behavioral strategies for managing conflict [2], [6], [18], [21]. Neither impartial intervention (policing) nor pacifying – two of these strategies – falls easily under the supposed catch-all cost-benefit definition of punishment. Policing and pacifying vary in cost to performer and in cost to target. In both cases, the direct cost (e.g. aggression received during the intervention) to target and performer can be close to zero, and the average cost of policing paid by powerful individuals is nearly zero [6], which violates common assumptions in punishment models. Policing and pacifying also vary on three rarely considered factors relating to benefits. They vary in their effectiveness at controlling the proliferation of aggression and reducing the frequency with which conflicts are expressed as fights. They also vary in terms of the demands made on social structure, with policing requiring rather special resource distributions or power structures to be accessible, regardless of whether individuals acquire them through learning or genetic inheritance. They are also likely to vary in indirect effects that operate over longer timescales, but little is known about this.

The theory literature on punishment makes a distinction between *peer punishment* and *pool punishment* [29]–[31]. In peer punishment individuals impose a fine on defectors at a cost to themselves. In pool punishment individuals contribute, prior to the joint effort, resources to a pool that funds defector control mechanisms like punishment. A “police force” paid for by taxes is an example. Hence pool punishment would appear to be distinguished from peer punishment by two factors: the assignment of conflict management roles to specific individuals or subgroups and a tax on the population to support these roles.

Our data, however, suggest that a tax levied specifically to support the role division is not necessary. To understand why this is the case, first consider how the policing role is assigned in our study system. The high variance power structure that supports policing emerges from status signaling network in which individuals give subordination signals to others they perceive to be more capable of using force [26]. The decision to signal is the outcome of an agonistic interaction history between the signal receiver and sender in which the sender has learned it is likely loose with that particular receiver. Conceding to the subordinate role by signaling is costly, but it is *less costly* than not signaling at all when an asymmetry in fighting ability is apparent [32]. It also has benefits as pairs with subordination contracts show increased socio-positive interactions over those without subordination contracts [32], [33]. Hence subordination signal exchange is in the interest of sender as well as receiver. As such the signaling dynamics are *cost-free* [34] as long as the subordination contract can be reversed if the underlying asymmetry in fighting ability is reversed, or terminated if the underlying asymmetry shrinks [27], [32].

Receivers by tracking the total number of signals they receive as well as how much agreement there is the number of signals sent by each sender can estimate how much power group members perceive them to have [6], which in turn tells them about the cost they will pay for intervening and engaging in social interactions more generally [26]. In many respects the signaling dynamics underlying the emergence of policing are like voting dynamics [32], as group members are, by virtue of how they distribute their signals, effectively determining whether there will be a “police force” and who should be on it. The policing role is in essence assigned to individuals through this voting scheme. This means that a police force can arise naturally if there already are pre-existing underlying heterogeneities in state (in our case, fighting ability or resource holding potential) that support signaling patterns leading to a heavy-tailed distribution of power [6]. No additional taxation is required beyond that which weaker individuals pay to maintain subordination contracts.

Looking forward, evolutionary models focusing on the function of policing will ideally derive optimum parameter values for intervention and switching based on empirically observed strategies. In this way frequency-dependent decisions involving policing will build upon a demonstrated density dependent dynamics of contagion.

### Future Work

The creation of an immuno-dynamic theory of conflict raises many issues for social systems. In much the way that there are optimal schedules for delivering drugs [35] that minimize opportunities for the evolution of resistance, are there schedules of intervention behavior that reduce co-evolutionary-escalation? Analyses quantifying strategic periodicities in conflict dynamics show that in our study group policing occurs on the hour timescale [24]. This suggests that the concept of intervention schedules in social systems is not farfetched. Often disease is not caused by infection but by an over-reactive immune system - immuno-pathology [36], [37]. This is reminiscent of the response of states to terrorism in which the principal damage is achieved by a response incommensurate with the magnitude of the attack. When policing has a high failure rate, this is what our models predict – anarchy or a police state. And there is in the intriguing phenomenon of immune memory, whereby, chronic low level infection might be required to ensure long term resistance to infection [38]. Does society require an analogous chronic conflict of low magnitude to maintain effective responses to rare, high-magnitude assaults? Whereas the analogy to the cellular immune system is only approximate, broad patterns of behavior associated with mechanisms for containing infection are expected to be rather general. By incorporating observations relating to individual variation, and individually-targeted responses, we foresee further parallels with the theory of clonal selection and expansion.

## Methods

### Ethics Statement

All data were collected in compliance with the ethical standards set by the Emory University animal care and welfare committee and IACUC approval (proposal 216-97). was obtained to conduct the study. As this was an observational study, the only change to the daily routine of the animals that was required to collect the data was that the animals had to be confined to their outdoor housing during each observation period. Water, monkey chow (remaining from morning feeding), enrichment (e.g. toys, climbing structures, etc.) and substantial space were available continuously throughout all observation periods. On very hot or rainy days, observations were terminated and the monkeys were given access to their indoor housing. As part of standard Yerkes management protocol, the animals were routinely subject to medical examination and care.

### Model System

Macaque societies are characterized by social learning at the individual level, social structures that arise from nonlinear processes and feedback to influence individual behavior, frequent non-kin interactions and multiplayer conflicts, the cost and benefits of which can be quantified at the individual and social network levels [7], [21]–[23], [39]. These properties coupled to highly resolved data make this system an excellent one for drawing inferences about critical processes in social evolution as well as for developing new modeling approaches that are intended to apply more broadly.

In this study we focus on one species in the genus, the pigtailed macaque (Macaca nemestrina). The data set, collected by J.C. Flack, is from a large, captive, breeding group of pigtailed macaques that was housed at the Yerkes National Primate Research Center in Lawrenceville, Georgia. Pigtailed macaques have frequent conflict and employ targeted intervention and repair strategies for managing conflict [23]. The study group had a demographic structure approximating wild populations. Subadult males were regularly removed to mimic emigration occurring in wild populations. The group contained 84 individuals, including 4 adult males, 25 adult females, and 19 subadults (totaling 48 socially-mature individuals used in the analyses). All individuals, except 8 (4 males, 4 females), were either natal to the group or had been in the group since formation. The group was housed in an indoor-outdoor facility, the outdoor compound of which was 125×65 ft.

Pigtailed macaques are indigenous to south East Asia and live in multimale, multifemale societies characterized by female matrilines and male group transfer upon onset of puberty [40]. Pigtailed macaques breed all year. Females develop swellings when in Œstrus.

### Data Collection Protocol

During observations all individuals were confined to the outdoor portion of the compound and were visible to the observer. The 156 hours of observations occurred for up to eight hours daily between 1,100 and 2,000 hours over a twenty-week period from June until October 1998 and were evenly distributed over the day. Provisioning occurred before observations, and once during observations. The data were collected over a four-month period during which the group was stable (defined as no reversals in status signaling interactions resulting in a change to an individual's power score [26]).

Conflict and power (subordination signal) data were collected using an all-occurrence sampling procedure [41] in which the compound was repeatedly scanned from left to right for onset of conflict or the occurrence of silent-bared teeth displays. The entire conflict event was then followed and data collected included start time, end time, the identity of individuals involved as aggressors, recipients, or interveners and their behavior.

### Operational Definitions

Conflict: any interaction in which one individual threatens or aggresses a second individual. A conflict was considered terminated if no aggression or withdrawal responses (fleeing, crouching, screaming, running away, submission signals) occurred for two minutes from the last such event. A conflict can involve multiple pairs if pair-wise conflicts result in aggressive interventions by third parties or redirections by at least one conflict participant.

Intervention: Third-party to conflict approaches with 3 m and directs aggressive behavior, affiliative behavior, submissive behavior, interposes itself between/equidistant to conflict participants, or approaches in a directed manner, looking at the conflict, but showing no other behavior.

Pacifying Intervention: Third-party to conflict approaches within 3 m and directs non-aggressive behavior at one conflict participant within 5 s of conflict. Nonaggressive behavior can include grooming, lip-smacking, puckering, presenting (directing hindquarters at another individual), or emitting a silent bared-teeth display.

Policing Intervention: Third-party to conflict approaches within 3 m and impartially threatens all conflict participants or interposes itself approximately equidistant to conflict participants within 5 s of conflict.

Cost: highest level of aggression received by an individual from any conflict participants in response to its intervention regardless of whether the conflict participant was the target of the intervention. Aggression varies from facial threat to severe bites (threat = 1 point, lunge or brief chase = 2 points, long chase or slap = 3 points, grapple or wrestle = 4 points, bite less than 5 seconds = 5 points, and bite greater than 5 s = 6 points).

Effectiveness: Interventions were considered effective if within 5 seconds of the intervention the entire fight was terminated, meaning that all conflict participants dispersed, or the intensity of aggression used by any of the participants was reduced (and remained reduced for the duration of the fight) without a concomitant increase in aggression or agitation (screaming) by any other participants.

Power: Degree of consensus among group members than an individual can use force successfully. Power scores for each individual were calculated using a procedure described in [26]. In brief, the total frequency of peacefully-emitted subordination signals (which reflect perception by the sender that the receiver can successfully use force [32]) received by an individual over a given duration (in this case, the study duration, which was approximately four months) is corrected for the uniformity (measured using Shannon entropy) of its distribution of signals received from its population of potential senders (all socially-mature individuals).

### Calculation of Corrected Frequencies

Raw data for all dependent variables (policing and pacifying frequency, cost, and effectiveness) were processed into deviation from expected frequencies (called observed minus expected scores previously, for full exposition see: [6]. This approach controls for underlying variation in the tendency to intervene.

### Model Details

In the following 6 models we introduce the stoichiometry and dynamical systems describing conflict management introduced in the paper. For the basic antibody model, and T cell model, we only present the stoichiometry since the analysis appears in the paper. For the remaining models, we provide details of stoichiometry, dynamics and dynamical stability. Throughout, parameters are as described in the paper.

### Antibody Model



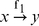
(13)

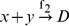
(14)

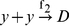
(15)

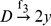
(16)

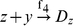
(17)

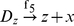
(18)


The initial conditions corresponding to the start of observations of behavior are, 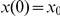, 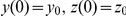 and 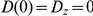.

### T cell Model

In the T cell model the conflict managers, called policers, do not target aggressives 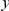 before they engage in conflict as in the antibody model, but resolve disputes directly, by intervening and eliminating fights between pairs forming in this case a triadic variable 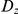. The kinetics of T cell conflict management are described by the following scheme:
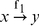
(19)

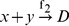
(20)

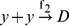
(21)

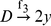
(22)

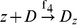
(23)

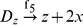
(24)


### Spontaneous Policing

Now instead of assuming a constant population of policers, we assume that passives can become police at some small rate. In addition, there is a small probability that policing fails to resolve fights in a satisfactory manner. In that case, instead of two passives and a police emerging from the interaction, all participants become aggressive. Thus the number of police is determined by the balance between the spontaneous new policing activity and the corruption of police in failed interventions. The system can be represented by the following “chemical” scheme and differential equations:
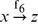
(25)

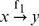
(26)

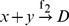
(27)

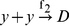
(28)

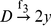
(29)

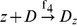
(30)

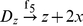
(31)

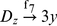
(32)


Assume, 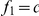, 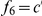, 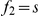, 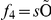, 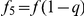, 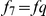 and 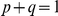. Here 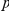 is the probability that a policing is successful.
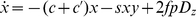
(33)

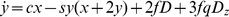
(34)

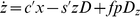
(35)

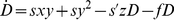
(36)

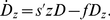
(37)


At steady state, we have 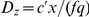, 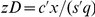, 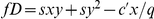. Substituting into the equation for 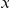, we get 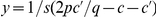. This is true if 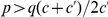, otherwise 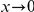 and hence 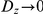 and 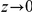 and the population consists entrirely of 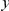
s and 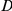
s.

We then obtain 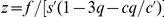, for which we require 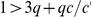. If this is the case then, since 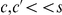, 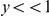 and 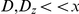 and hence 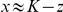. Otherwise, we get 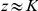.

Thus there are three regimes: 1) If 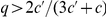, then the steady state population consists almost entirely of 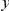
s and 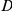
s. 2) If 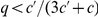, then the steady state population consists of 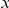
s and 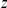
s with 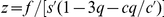. 3) If 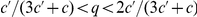 then the steady state populations consists almost entirely of 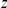
s.

Since we assume that 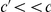, the value of 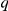 must be small in order to avoid conflict.

### Temporary Aggression

We now study a variant of the previous model in which aggressives become passive at some slow rate. The model can be represented:
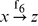
(38)

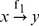
(39)

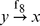
(40)

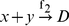
(41)

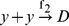
(42)

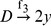
(43)

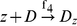
(44)

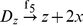
(45)

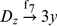
(46)


Assume, 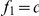, 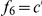, 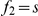, 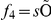, 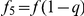, 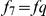, 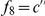 and 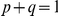.
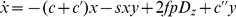
(47)

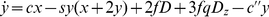
(48)

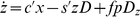
(49)

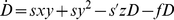
(50)

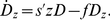
(51)


This system has two steady states, namely when the whole population consists of passives and when it consists of police. The all 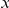 state is unstable and the all 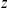 state is stable. Thus in this version of the model for which aggressives spontaneously become passive at some slow rate, and passives spontaneously become police at some slow rate, the population evolves to a state in which almost the whole population consists of police.

### Conditional Policing

We now remove the assumption that the aggressive population spontaneously become passive and add the assumption that if police encounter other police, one of them stops policing and becomes passive. The rationale is that police only want to incur the cost of policing if they don't believe that another individual will police. This is a form of negative frequency-dependence.

The system can be represented:
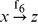
(52)

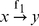
(53)

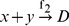
(54)

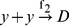
(55)

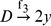
(56)

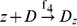
(57)

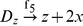
(58)

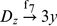
(59)

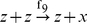
(60)


Assume, 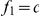, 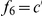, 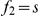, 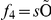, 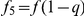, 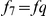, 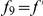 and 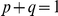.
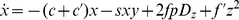
(61)

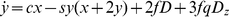
(62)

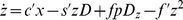
(63)

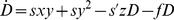
(64)

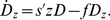
(65)


This has two steady states, namely all 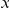 and all 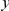 and 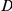. This is true unless 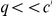, in which case the all 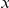 state is replaced by a state in which 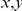 and 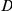 are all present. The all 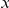 state is unstable while the all 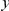 and 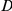 state is stable.

### Conditional, Temporary Policing

In this case aggressive individuals can spontaneously become passive and two police interact to give one police and one passive. The system is represented:
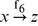
(66)

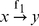
(67)

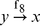
(68)

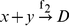
(69)

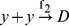
(70)

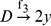
(71)

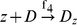
(72)

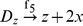
(73)

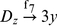
(74)

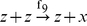
(75)


Assume, 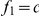, 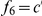, 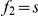, 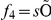, 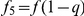, 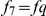, 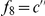, 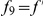 and 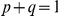.
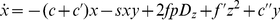
(76)

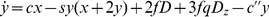
(77)

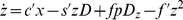
(78)

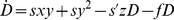
(79)

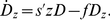
(80)


This has a single steady state with
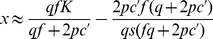
(81)

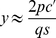
(82)and the rest adopt strategy 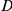.

This assumes that 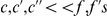 but that 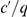 may be reasonably large.

Thus in a society in which the police remain vigilant in the face of policing, the aggressive state is of finite duration, and the steady state consists of a mixture of passive, aggressive, and fighting populations with very few police.
